# Ultra-Fast Intraoperative *IDH*-Mutation Analysis Enables Rapid Stratification and Therapy Planning in Diffuse Gliomas

**DOI:** 10.3390/ijms26199639

**Published:** 2025-10-02

**Authors:** Theo F. J. Kraus, Beate Alinger-Scharinger, Celina K. Langwieder, Anna Mol, Tereza Aleksic, Brain van Merkestijn, Hans U. Schlicker, Mathias Spendel, Johannes Pöppe, Christoph Schwartz, Christoph J. Griessenauer, Karl Sotlar

**Affiliations:** 1 Institute of Pathology, University Hospital Salzburg, Paracelsus Medical University, Müllner Hauptstraße 48, A-5020 Salzburg, Austria; 2 Department of Neurosurgery, University Hospital Salzburg, Paracelsus Medical University, Ignaz-Harrer-Straße 79, A-5020 Salzburg, Austria

**Keywords:** glioma, glioblastoma, *IDH* mutation, intraoperative genetics, integrated diagnosis

## Abstract

Diffuse gliomas are the most common primary brain tumors in adults in the Western world. According to the 2021 World Health Organization (WHO) classification of central nervous system (CNS) tumors, the assessment of isocitrate dehydrogenase (*IDH1/2*)-mutation status is essential for accurate patient stratification. In this study, we performed a comprehensive evaluation of *IDH*-mutation status in the intraoperative setting using the Idylla platform. The reference cohort comprised 30 formalin-fixed paraffin-embedded (FFPE) tissue samples with known *IDH* status, while the exploration cohort included 35 intraoperative snap-frozen and native-tissue specimens. The results were compared with those of a standard next-generation sequencing (NGS) analysis. Our findings demonstrate that the Idylla *IDH*-mutation assay provides 100% concordance compared with NGS analysis for both FFPE and intraoperative tissue samples. The Idylla system delivers results within approximately 90 min, significantly outperforming NGS, which requires between 7 and 27 days. This rapid turnaround facilitates timely interdisciplinary case discussions and enables timely therapy planning, within the framework of neuro-oncological molecular tumor boards. The ultra-fast intraoperative *IDH*-mutation analysis using the Idylla platform, in combination with intraoperative histopathological assessment, enables rapid patient stratification and treatment planning in diffuse gliomas.

## 1. Introduction

Gliomas constitute a heterogeneous group of primary brain tumors that originate from glial cells [1]. These neoplasms comprise approximately 30% of all central nervous system tumors and 80% of all malignant brain tumors [1,2]. The classification of these tumors is combined by integrating histological features and molecular genetic hallmarks [1]. Thereby, a substantial enhancement in the comprehension of glioma biology has been achieved through the identification of mutations in the isocitrate dehydrogenase (*IDH*) genes, particularly *IDH1* and *IDH2* [1]. These mutations are identified in astrocytoma as well as oligodendroglioma and are thus established as critical biomarkers for both diagnosis and prognosis [1,3,4]. The *IDH1* mutations, which occur at the R132 position, result in the generation of 2-hydroxyglutarate (2-HG), an oncometabolite, in addition to the physiologically present α-ketoglutarate (αKG) [5,6,7]. The accumulation of 2-HG has been implicated in the promotion of glioma genesis [5,8,9]. In addition, *IDH*-mutation status is associated with patient outcomes in diffuse gliomas: patients with *IDH*-mutated gliomas, i.e., astrocytomas and oligodendrogliomas, tend to have longer overall survival compared with those with IDH-wildtype gliomas, i.e., glioblastomas [1,10,11]. While astrocytomas only show *IDH* mutations, oligodendroglioma shows coexistence of *IDH* mutations along with additional losses of chromosome 1p and 19q (LOH1p/19q) [1].

After definite integrated glioma diagnosis, patients are treated in an individualized setting including adjuvant radio-chemotherapy with temozolomide according to the EORTC/NCIC protocol [12]. Thereby, patients with O6-methylguanine-DNA methyltransferase (*MGMT*) promoter methylation show a better therapy response compared with patients with an unmethylated *MGMT* promoter [13,14,15]. Thus, the revelation of the *IDH*-mutation status remains the main hallmark in patient stratification and interdisciplinary board-certified therapy planning [1]. This is of particular interest with the introduction of IDH inhibitors as a viable treatment option for *IDH*-mutated low-grade gliomas [16,17].

Thereby, the prognostic significance of *IDH* mutations emphasize the necessity for the development of rapid and reliable diagnostic methods for their detection. Conventional methods, such as Sanger sequencing, next-generation sequencing (NGS), and digital droplet PCR (ddPCR), that are frequently used in clinical practice are hands-on and time intensive [1,11,18].

Thus, there is clinical need for the rapid and accurate identification of *IDH* mutations that enables optimal practice in clinical environments where prompt treatment decision findings are imperative. This, in turn, has the potential to enable early intervention and the development of personalized treatment strategies [19,20,21,22].

The Idylla system enables fast and automated real-time PCR-based analysis of distinct mutations in various genes, e.g., B-Raf proto-oncogene serine/threonine kinase (*BRAF*) in various cancers [23], Kirsten rat sarcoma viral oncogene homolog (*KRAS*) and neuroblastoma RAS viral oncogene homolog (*NRAS*) in colorectal cancer [24], and epidermal growth factor receptor (*EGFR*) in non-small cell lung cancer [25], and even the detection of mismatch-repair deficiency (dMMR) in endometrial cancer, colorectal cancer, and gastric adenocarcinomas [26,27,28]. The Idylla system performed DNA extraction, quantitative PCR, and mutation detection as well as the interpretation of results in a fully automated setting [23,24,25,26,27,28]. Thus, the Idylla system is ideal for fast molecular genetic testing in the clinical setting.

In this study, we performed an ultra-fast intraoperative molecular genetic stratification of the IDH-mutation status in glioma, applying the Idylla system, and validated the results with conventional NGS-based analysis [29,30,31,32,33,34].

## 2. Results

### 2.1. Idylla IDH Mutation Assay Delivers Accurate Results on FFPE Tissue

The Idylla *IDH*-mutation assay system, according to the manufacturer, is dedicated to the analysis of FFPE tissue. Therefore, the initial step was to establish a system for analyzing FFPE glioma tissue samples with known *IDH*-mutation status. The *IDH* status was assessed by applying NGS in the current 2021 routine diagnostic setting. For the reference cohort, a total of 30 cases with known IDH-mutation status were selected: 15 cases each from the *IDH*-wildtype and *IDH*-mutation groups, respectively. For a more thorough examination of the sample, please refer to Supplementary Table S1.

**Figure 1:**
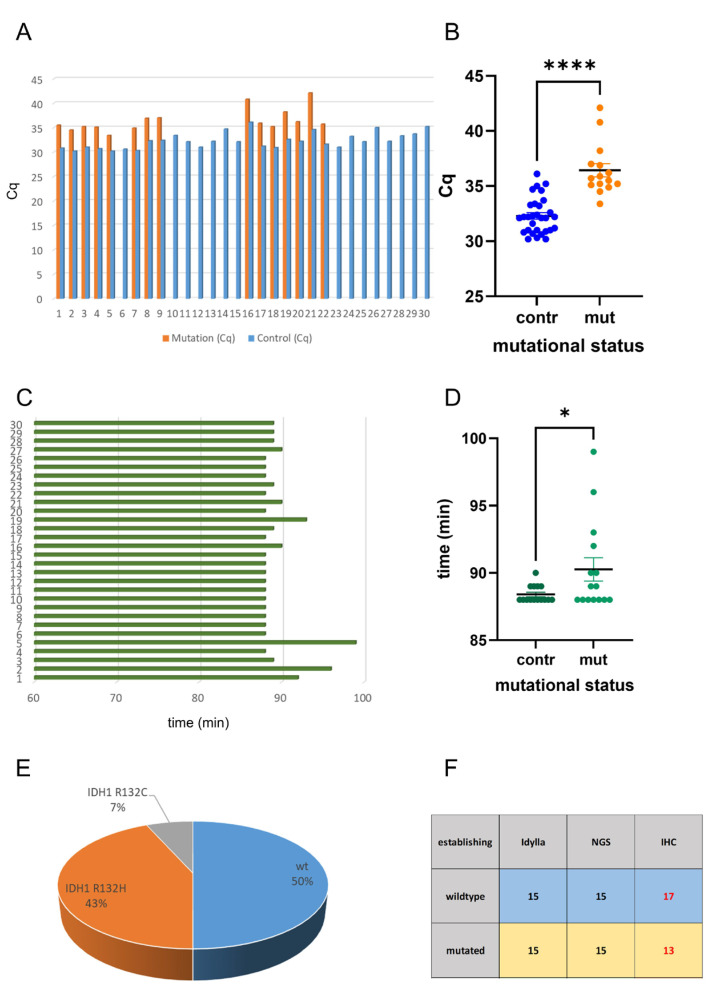
Analysis of IDH mutations in the reference cohort. The Idylla system was applied in 30 FFPE cases with known IDH-mutation status. Indicated are the Cq values of the analysis of wildtype and mutated samples (**A**). Statistical analysis showed that the Cq values of the controls were significantly lower than the Cq values of the mutations (**B**). Indicated are the elapsed time periods of the analysis (**C**). Statistical analysis showed that the results of the wildtype samples were obtained significantly earlier than those of the mutated samples (**D**). NGS showed 15 samples with *IDH1/2* wildtype, and 15 samples were *IDH1* mutated: 13 samples showed the *IDH1* R132H mutation and 2 samples showed the *IDH1* R132C mutation) (**E**). While Idylla showed 100% concordance with NGS, the *IDH1* R132H-mutation-specific antibody missed 2 mutated cases (**F**). * *p* < 0.05; **** *p* < 0.0001. red labels: discordant results.

The Idylla system detected mutations in all 15 cases: consequently, the Cq values of the control regions were consistently identifiable prior to the mutations, i.e., the Cq values of mutation detection were elevated (Figure 1A). A statistical analysis revealed that the Cq values of the controls were significantly lower than the Cq values of the mutations (Figure 1B). The analysis of the obtained results indicates that the wildtype samples exhibited a faster response time in comparison with the mutated samples (Figure 1C). A statistical analysis revealed that the results of the wildtype samples were obtained significantly faster than those of the mutated samples (Figure 1D). This phenomenon may be attributed to the presence of additional controls performed in mutated cases. The mutation status of all samples was known based on NGS data: 15 samples were of *IDH1/2* wildtype, and 15 samples were *IDH1* mutated (13 samples showed the *IDH1* R132H mutation, and 2 samples showed the *IDH1* R132C mutation) (Figure 1E). There were no cases with *IDH2* mutations available, due to the low abundance of these mutations. The Idylla system showed 100% concordance with NGS data: all 15 wildtype and all 15 *IDH1* mutated cases were correctly identified (Figure 1F). In contrast to NGS and the Idylla system, immunohistochemistry revealed only 13 of the 15 *IDH1* mutated cases. This is because the antibody only recognizes the *IDH1* R132H mutated protein (Figure 1F).

The implementation of the Idylla system has effectively demonstrated the capacity to methodically identify *IDH* mutations within formalin-fixed, paraffin-embedded (FFPE) tissue.

### 2.2. Analysis of Intraoperative Snap-Frozen and Fresh Samples Delivers Fast and Valid Results When Applying the Idylla IDH-Mutation Assay

Subsequently, we conducted Idylla *IDH*-mutation analysis on intraoperative snap-frozen and fresh-tissue samples. Tissue samples were obtained during neurosurgical procedures, specifically in the context of sectioning but also in stereotactic biopsy settings. The standard workup procedure entailed the generation of squash sections and snap-frozen sections. Following the completion of the histological evaluation, two to five sections of tissue, each measuring 10 µm, were subjected to Idylla *IDH*-mutation analysis. This exploration cohort comprised 35 cases. Further details can be found in Supplementary Table S2.

**Figure 2:**
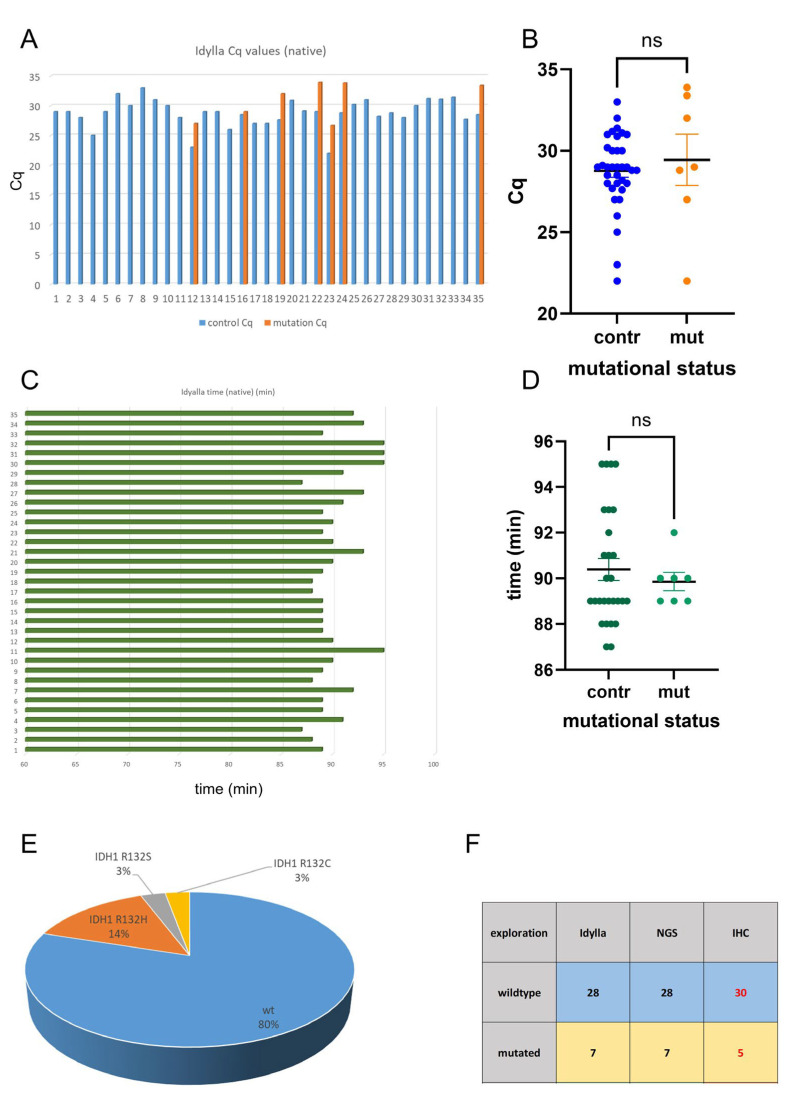
Analysis of *IDH* mutations in the exploration cohort. The exploration cohort consisted of 35 snap-frozen and native samples. Indicated are the Cq values of the control regions and the mutations (**A**). Statistical analysis showed no difference in the Cq values of the control regions and the mutations (**B**). Indicated are the elapsed time periods of the analysis (**C**). There was no statistically significant difference in the analysis time of the mutated and wildtype samples (**D**). NGS analysis of all 35 samples showed that 28 samples were *IDH1/2* wildtype; seven cases were *IDH1* mutated: five showed the *IDH1* R132H mutation, one showed the *IDH1* R132C mutation, and one case showed the IDH1 R132S mutation. There was no *IDH2* mutated case (**E**). While Idylla showed high concordance with NGS in the current small cohort, the *IDH1* R132H-mutation-specific antibody missed 2 mutated cases (**F**). n.s. not significant. red labels: discordant results.

Of the 35 cases examined, 7 exhibited mutant *IDH* status. The Cq values of the control regions were consistently identifiable prior to the mutations (Figure 2A). A subsequent analysis revealed no statistically significant difference between the Cq values of the control regions and the mutations (Figure 2B). All Idylla analyses were completed within 95 min (Figure 2C). There was no statistically significant difference in analysis time of the mutated and wildtype samples (Figure 2D). According to the results of the Idylla analysis, of the 35 cases, 28 were of *IDH1/2* wildtype status and 7 were *IDH1* mutated. There was no case with *IDH2* mutation. The mutation status of all 35 samples was verified by NGS: 28 samples were IDH1/2 wildtype, 7 cases were *IDH1* mutated; there was no IDH2 mutated case. Of the *IDH1* mutated cases, five showed the *IDH1* R132H mutation, one showed the *IDH1* R132C mutation, and one case showed the *IDH1* R132S mutation (Figure 2E). In the small cohort analyzed in this study, the Idylla system showed perfect concordance with NGS data (Figure 2F); however, this finding should be validated in larger cohorts. In contrast to NGS and the Idylla system, immunohistochemistry revealed only five out of seven *IDH1* mutated cases. Two cases were not detected (*IDH1* R132C and *IDH1* R132S) (Figure 2F).

Thus, the Idylla system is a highly valid technique in the identification *IDH* mutation of even fresh-frozen and native-tissue samples.

### 2.3. Idylla IDH-Mutation Analysis Enables Ultra-Fast and Reliable IDH-Mutation Detection Compared with Routine NGS Analysis

**Figure 3:**
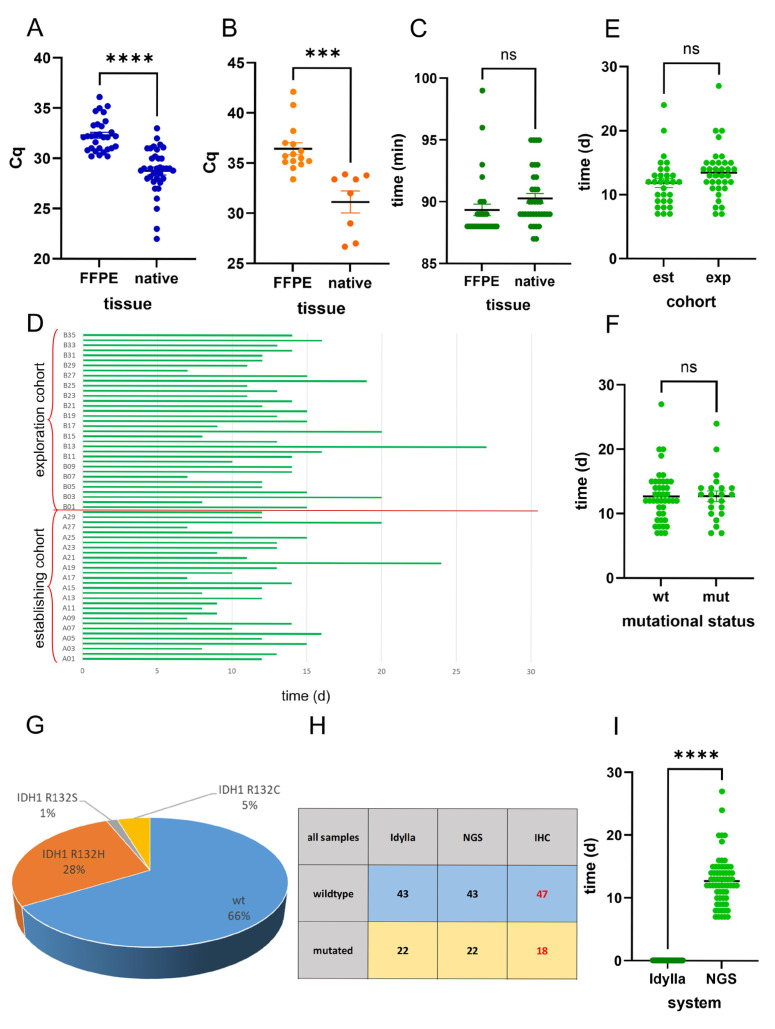
Comparison of the results of FFPE and native tissue. A comparison of the Cq values showed that the Cq values were significantly lower in the snap-frozen and native samples compared with the FFPE samples in wildtype (**A**) and mutated samples (**B**). A comparison of the elapsed time in the two cohorts showed that results were obtained within a time frame of less than 100 min (**C**). Analysis of the time elapsed in the routine NGS analysis showed a period ranging from 7 to 27 days (**D**). There was no statistically significant difference in the NGS analysis times of the retrospective establishing and prospective exploration cohorts (**E**). In addition, there was no statistically significant difference in the NGS analysis time of *IDH*-mutated and *IDH*-wildtype cases (**F**). Of the *IDH1* mutated cases, 18 were *IDH1* R132H, 3 were R132C, and 1 was R132S mutated (**G**). All mutations were detected correctly by the Idylla system, but immunohistochemistry missed four cases (**H**). Comparing the time elapsed from surgery to the results of the IDH-mutation analysis, the mean NGS analysis time was 12.71 days, in contrast to the mean analysis time of 89.84 min achieved using the Idylla *IDH*-mutation system (**I**). n.s. not significant, *** *p* < 0.001; **** *p* < 0.0001. red labels: discordant results.

Next, we compared the results of FFPE and native tissue. A comparison of the Cq values revealed that the Cq values were significantly lower in the snap-frozen and native samples compared with the FFPE samples, both in the wildtype (Figure 3A) and mutated (Figure 3B) samples.

A comparison of the elapsed time from the start of the analysis to the output of the mutation results in the two cohorts was conducted: the Idylla *IDH*-mutation system provides rapid results regarding the *IDH*-mutation status of both FFPE and native samples, within a time frame of less than 100 min (Figure 3C).

Analysis of the time elapsed in routine NGS analysis showed a period ranging from 7 to 27 days (Figure 3D). There was no statistically significant difference in the NGS analysis times of the retrospective establishing and prospective exploration cohorts (Figure 3E). In addition, there was no statistically significant difference in the NGS analysis time of *IDH*-mutated and *IDH*-wildtype cases (Figure 3F).

Summary of both cohorts showed that of 65 cases, 43 were of *IDH1/2* wildtype status, and 22 showed *IDH1* mutations. There was no case with *IDH2* mutation. Of the *IDH1* mutated cases, 18 were *IDH1* R132H, 3 were R132C, and 1 was R132S mutated (Figure 3G). All mutations were detected correctly by the Idylla system (Figure 3H). Immunohistochemistry revealed only 18 mutated cases (only *IDH1* R132H mutated cases) (Figure 3H). Comparing the time elapsed from surgery until the results of the *IDH* mutation analysis, the mean NGS analysis time was 12.71 days, in contrast to the mean analysis time of 89.84 min achieved using the Idylla *IDH*-mutation system (Figure 3I).

In summary, the Idylla *IDH*-mutation system delivers ultra-fast and reliable results of both FFPE and snap-frozen/native-tissue samples in the setting of routine and intraoperative histological assessment with 100% concordance with routine NGS diagnostics.

## 3. Discussion

Gliomas constitute a heterogeneous group of primary brain tumors, accounting for approximately 30% of all central nervous system (CNS) neoplasms and 80% of malignant brain tumors [1,2]. Their classification is based on an integrated approach that combines morphological features with molecular markers [1,35]. Among these markers, the mutational statuses of isocitrate dehydrogenase genes *IDH1* and *IDH2* are of particular relevance, as they demarcate biologically distinct glioma entities with profound prognostic and therapeutic implications [4,17,36,37].

According to the 2021 WHO classification of CNS tumors, the differentiation of high-grade gliomas lies in the molecular stratification between Grade 4 *IDH*-mutant astrocytomas and conventional grade 4 *IDH*-wildtype glioblastomas [1]. *IDH*-mutant astrocytomas, although histologically assigned WHO grade 4 due to features such as microvascular proliferation and necrosis, exhibit a markedly different clinical trajectory compared with their *IDH*-wildtype counterparts [1]. These tumors tend to arise in younger patients, progress more slowly, and are associated with significantly longer overall survival [1]. The presence of an *IDH* mutation leads to the production of the oncometabolite 2HG, which induces widespread epigenetic reprogramming and contributes to a distinct tumor microenvironment [5,6,7]. In contrast, *IDH*-wildtype glioblastomas represent the prototypical and most aggressive form of adult glioma, often presenting de novo in older individuals with rapid clinical deterioration [1]. These tumors lack the metabolic and epigenetic alterations conferred by *IDH* mutations and are instead characterized by a high degree of genomic instability [1]. The divergent molecular landscapes between *IDH*-mutant and *IDH*-wildtype grade 4 gliomas have led to their separation into distinct diagnostic entities in the WHO CNS tumor classification, reflecting not only their biological heterogeneity but also their differing responses to therapy and prognostic implications [1]. This molecular distinction underscores the importance of integrated histopathological and genomic profiling in guiding personalized treatment strategies and accurately predicting disease course [1].

Following a definitive integrated diagnosis of glioma, therapeutic management is tailored to the individual patient and typically involves adjuvant radio-chemotherapy with temozolomide, administered in accordance with the standardized EORTC/NCIC protocol [12]. A critical molecular determinant of treatment efficacy within this regimen is the methylation status of the *MGMT* gene promoter. *MGMT* encodes a DNA repair enzyme that counteracts the cytotoxic effects of alkylating agents such as temozolomide by removing alkyl groups from the O6 position of guanine, thereby mitigating DNA damage-induced apoptosis. Promoter methylation of the *MGMT* gene leads to transcriptional silencing and reduced expression of the repair enzyme, resulting in increased sensitivity of tumor cells to temozolomide-induced DNA lesions. Consequently, patients whose tumors exhibit *MGMT* promoter methylation tend to experience significantly improved therapeutic responses, including prolonged progression-free and overall survival, compared with those with unmethylated *MGMT* promoters, who often demonstrate resistance to alkylating chemotherapy [13,14,15]. This epigenetic biomarker has thus emerged as a pivotal prognostic and predictive factor in the clinical decision-making process for glioma treatment, guiding both therapeutic stratification and expectations regarding treatment outcomes.

*IDH* mutations typically involve *IDH1* R132H and less frequently IDH2 R172K [1,36,38]. Accumulating evidence indicates that *IDH*-mutant gliomas have a relatively prolonged progression-free and overall survival compared with *IDH*-wildtype tumors [39,40,41,42]. Consequently, the determination of *IDH*-mutation status is not merely a diagnostic criterion but a crucial prognostic and predictive biomarker.

Despite its relevance, traditional methods for *IDH*-mutation detection, such as Sanger sequencing, pyrosequencing, and next-generation sequencing (NGS), entail technical complexity, high costs, and relatively long turnaround times compared with immunohistochemistry that does not reveal all relevant *IDH* mutations [43,44].

To address this need, rapid molecular diagnostic platforms have been developed. Among them, the Idylla system represents a fully automated, cartridge-based real-time PCR assay capable of detecting clinically relevant *IDH1/2* mutations directly from formalin-fixed paraffin-embedded (FFPE) or fresh tissue with minimal hands-on time [45]. The Idylla system was already successfully applied to detect gene mutations, such as *BRAF*, *KRAS*, *NRAS*, *EGFR*, and dMMR in various cancers, such as non-small cell lung, endometrial, colorectal, and gastric cancer [23,24,25,26,27,28]. Thus, the Idylla system is ideal for fast molecular genetic testing in the clinical setting.

In our study, we evaluated the performance of the Idylla *IDH*-mutation assay on intraoperatively obtained snap-frozen and native-tissue samples. Remarkably, the assay yielded results within approximately 90 min, offering a dramatic reduction in turnaround time compared with conventional sequencing techniques. The *IDH* mutation status determined by Idylla was 100% concordant with results obtained from reference NGS performed on matched FFPE samples.

Our findings align with and extend those of recent investigations. For example, the utility of real-time PCR and digital PCR approaches for the rapid identification of *IDH1* R132H in gliomas, reporting comparable sensitivity and specificity to sequencing-based methods, was demonstrated [1,11,18]. Moreover, immunohistochemistry (IHC) using mutation-specific antibodies, i.e., for *IDH1* R132H, remains a widely used, cost-effective alternative in many centers. However, IHC fails to detect rare non-canonical mutations, thus necessitating molecular confirmation in equivocal cases [1,43].

Other emerging technologies include pyrosequencing, digital droplet PCR (ddPCR), nanopore sequencing, and mass spectrometry-based methods, which offer high sensitivity for low-frequency variants and compatibility with small sample volumes [20,46,47,48,49,50,51]. While promising, these techniques typically require specialized infrastructure and technical expertise, limiting their intraoperative feasibility [20,46,47,48,49,50]. In contrast, the Idylla system operates as a closed, user-friendly platform requiring minimal training and offering standardized output—making it particularly attractive for integration into neuro-oncological workflows [45].

Incorporating such rapid assays into intraoperative decision-making processes has the potential to enhance surgical and therapeutic strategies. For instance, in *IDH*-wildtype tumors with aggressive behaviors, maximal gross total resection may be prioritized, whereas in *IDH*-mutant gliomas, resection volume may be adjusted based on biological profile [16,52,53,54,55]. Furthermore, early molecular stratification may facilitate enrollment into clinical trials that increasingly mandate rapid molecular data as inclusion criteria [16,52,53,54,55].

Our study underscores the feasibility and clinical utility of ultra-rapid *IDH*-mutation testing using the Idylla platform on fresh and native glioma tissue. By providing reliable molecular results within 90 min, this approach enables early, personalized patient management and bridges a critical gap in current diagnostic workflows. Future studies should investigate its applicability to additional biomarkers, such as telomerase reverse transcriptase (*TERT*) mutation, and 1p/19q co-deletion, to further expand the armamentarium of real-time precision diagnostics in neuro-oncology. Additionally, there are approaches to predict the *IDH* status by radiological means [56,57,58,59,60]. However, the definitive proof of *IDH* status is proven by molecular analysis [1].

In conclusion, the Idylla *IDH*-mutation system is an ultra-fast and highly reliable, valid method for detecting *IDH* mutations in gliomas, enabling fast and accurate individualized patient care.

## 4. Materials and Methods

### 4.1. Tissue Collection

We analyzed a total of 65 cases. Cases were allocated to two distinct cohorts: a reference cohort and an exploration cohort. The reference cohort, which served to establish the system, contained 30 formalin-fixed and paraffin-embedded tissue (FFPE) samples with already known *IDH*-mutation status. The exploration cohort consisted of 35 native and snap-frozen samples. Classification of gliomas was performed in accordance with the current 2021 CNS WHO classification [1]. Immunohistochemistry using the mutation-specific *IDH1* (R132H) antibody (Dianova Biozol Diagnostica, Hamburg, Germany) was performed according to the manufacturer’s protocol on a Ventana Bench Mark Ultra stainer (Roche, Basel, Switzerland) as reported previously [29,30,31].

### 4.2. Molecular Genetic Characterization of Gliomas

Reference molecular genetic analysis of glioma samples was performed as previously described [23,26,27,28]. DNA extraction was performed, applying the Maxwell system (Promega, Madison, WI, USA) according to the manufacturer’s instructions [29]. A mutational analysis of the *IDH1* and *IDH2* genes was conducted using the AmpliSeq for Illumina Focus Panel (Illumina, San Diego, CA, USA). These analyses were performed on an Illumina MiniSeq next-generation sequencing device in accordance with the manufacturer’s protocols [29].

The ultra-fast genetic analysis of *IDH1* and *IDH2* was performed using the Idylla system (Biocartis, Mechelin, Belgium) and the Idylla *IDH1-2* Mutation Assay Kit (Biocartis, Mechelin, Belgium). The assay detects the most prevalent 15 mutations of *IDH1/2* genes: *IDH1* R132C/H/G/S/L, *IDH2* R140Q/L/G/W, and *IDH2* R172K/M/G/S/W. The Idylla system and the *IDH1-2* Mutation Assay Kit were utilized in accordance with the manufacturer’s protocol. The single-use 50 µL DNA oligos containing assay-specific reagents for qPCR, i.e., allele-specific primers and probes to detect the 15 designated *IDH1* and *IDH2* mutations, was added directly to the reaction cartridge. Tissue sections (FFPE or native/snap frozen) were placed between two filter papers that had been wet with 100 µL of nuclease-free water. These sections were then added directly to the reaction chamber of the Idylla cartridge. Deparaffination, DNA extraction, and quantitative polymerase chain reaction (qPCR) were performed in the single-use cartridges that contained all necessary reagents. The results were obtained after a runtime of approximately 1.5 h. The results report contained the following information: cartridge ID, oligo ID, and sample ID; assay start and stop time; results of the mutation analysis, including the calculated cycle of quantification (Cq) of controls and mutations; and assay quality information (i.e., the validity of results).

### 4.3. Statistical Data Analysis

Statistical analysis was performed using the GraphPad Prism (version 10) software suite and Microsoft Excel applying Student’s *t*-test. Statistical significance was assumed for *p*-values < 0.05.

## Data Availability

The original contributions presented in this study are included in the article/Supplementary Material. Further inquiries can be directed at the corresponding author(s).

## Supplementary Materials

The following supporting information can be downloaded at https://www.mdpi.com/article/10.3390/ijms26199639/s1.

## Abbreviations

The following abbreviations are used in this manuscript:

LGG	low-grade glioma
HGG	high-grade glioma
GBM	glioblastoma
IDH	isocitrate dehydrogenase
αKG	α-ketoglutarate
2-HG	2-hydroxyglutarate
LOH1p/19q	loss of heterozygosity of chromosome 1p and 19q
MGMT	O6-methylguanine-DNA methyltransferase
NGS	next-generation sequencing
ddPCR	digital droplet PCR
FFPE	formalin-fixed, paraffin-embedded
IHC	immunohistochemistry
BRAF	B-Raf proto-oncogene serine/threonine kinase
KRAS	Kirsten rat sarcoma viral oncogene homolog
NRAS	neuroblastoma RAS viral oncogene homolog
EGFR	epidermal growth factor receptor
dMMR	mismatch-repair deficiency
TERT	telomerase reverse transcriptase
